# To Junior Dentists

**Published:** 1886-06

**Authors:** 


### TO JUNIOR DENTISTS. NO. V.

### ALVEOLAR ABSCESS.

*My Dear Doctor* :

I am continually restricted by the poverty of our nomenclature, and yet, had I the vocabulary of the wordiest and most verbose man in existence, I do not know that I should be better off. The addition of terms and the multiplying of nominations only bring confusion to the student. There would be no end to the refinements of definition and the nice shades of distinction if once the rein be given to hair-splitting.

There are a number of different conditions, all included in the term alveolar abscess. Any pus-discharging sinus connected with the alveolus may properly be so called. It may be caused by the presence of a foreign substance, by an accidental concretion, by necrotic tissue, or by a diseased peridental membrane. It might be well to distinguish them, but it is not essential, and so I shall not attempt the invention of new names for old diseases. In what I am about to say I shall confine myself to that form of abscess caused by a septic condition of a tooth canal, which is so often the accompaniment of a devitalized pulp, and which is confined to the neighborhood of the apex of the tooth-root.

Were I to attempt to speak to you of alveolar abscess exhaustively, I must first consider the subject of inflammation, and this would involve a treatise on assimilation, circulation, innervation, and many other "ations." We must necessarily study all pathological changes as related to it, and this would involve the physiology of the system. We could not comprehend it without a thorough knowledge of the anatomy of the body, both general and minute, and the pathology would imply an acquaintance with materia medica and therapeutics. All cachectic conditions and dyscrasias have an influence on tissues, and the particular condition of any one organ may affect the other, so that it might be necessary, in any special instance, to consider the condition of the whole body, and to trace the interdependence of the several organs and tissues. This, you see, includes the whole practice of medicine, and it is a fact, that so intimately are interwoven the functions of the different parts, a full comprehension of one involves a study of all. When, therefore, any one asserts that a knowledge of general medicine is of no special value to a dentist, ask him if he thinks that the oral tissues have no connection with the rest of the body, and are not dependent upon other organs for their condition. We will not, however, go back to consider all the nutrient currents that may influence a dental condition, but take up just the subject in hand, and examine it by itself.

An ordinary alveolar abscess is the breaking down of tissue, and the disorganization of that pabulum or food which the system supplies to injured or diseased parts. Its initial point is the apex of the root of a tooth. It is a septic condition of the territory at the extremity of a tooth-socket, and its existence implies a dead pulp. An alveolar abscess cannot exist with a live pulp, for it is inconsistent to suppose that the pulp shall be nourished and the pulp vessels retain their integrity when passing through such a devastating fire as precedes the active stage of alveolar abscess.

It also implies that there was some communication between the disturbed territory and the outside world (as in the case of a compound fracture of a bone), for without this the microbes that cause, or at least attend the condition, could not find access. I do not say that a fistula may not form except there be a dead pulp, but if it does it will not be the alveolar abscess of which I am speaking. An abscess may also form in connection with an undecayed tooth containing a dead pulp, but that, too, will not be the ordinary alveolar abscess. Usually, you will find that when a pulp has died because of some blow or injury to the tooth, and without any exposure of the pulp or socket, no abscess ensues.

The ordinary course of alveolar abscess is this: When, through decay or by accident a pulp is exposed, it dies, and putrefaction of the tissues follows. The gases of this process pass through the foramen of the tooth and carry with them septic organisms, and thus induce a violent inflammation of the pericemental membrane, which goes through the various stages until suppuration is reached, and the pus, seeking the readiest outlet, breaks through the alveolar walls and a fistula is established. There cannot be putrefaction of the pulp and the evolution of offensive gases without the presence of putrefactive organisms, so that I believe I am right when I say that ordinary alveolar abscess cannot exist without the presence of a dead pulp and the penetration of micro-organisms. If the pulp-chamber be hermetically sealed, the pulp-tissue will dry up and desiccate. It will not produce alveolar abscess.

The septic condition, once established at the end of the root of a tooth, will usually be continued as long as the territory remains in the septic state. Nature will make attempts at a cure, but unaided, she will not usually succeed. There will be an effusion of plasmic matter about the point of disease, and an attempt to build this up into tissue, which, so long as this septic condition continues, will be abortive. A wall of plasma will surround the lesion, the outer part of which will be healthy lymph, while that nearest the diseased point will be broken down and disintegrated. It is this barrier of tissue-pabulum, thrown out by the system to repair the waste, which forms the so-called pyogenic membrane, the sac that is often extracted with an abscessed tooth.

An abscess does not, in all cases, immediately follow the death of even an exposed pulp. There are a number of reasons why this may not be the case :

1st. The point of exposure may be so wide that all the products of the putrefaction of pulp-tissue may find entrance into the mouth, and none pass through the foraminal opening.

2d. The vitality of the pericemental membrane may be so high as successfully to resist the septic organisms.

3d. The pulp-chamber and canals may become the seat of a kind of fungoid vascular growth, that precludes septic influence.

4th. The foramen may become closed, and thus prevent pericemental infection.

A tooth that has lost its pulp may exist for a long time without becoming the seat of an abscess, and this immunity may be due to either of the above causes, and yet that influence may cease eventually, when the abscess will ensue. An undecayed tooth, having a dead pulp which has given no trouble for years, immediately upon being drilled open may develop lively pericemental inflammation, which will result in an abscess. This is because an opportunity for the entrance of septic organisms has suddenly been given. Had the opening been made under aseptic conditions and no foreign matter or debris been allowed to pass the foramen, this inflammation would not have occurred. We must remember that an ordinary alveolar abscess implies a septic condition, and the presence of septic organisms.

You will remember that in my last letter I spoke to you of the importance of a clear conception of what the terms septic and aseptic mean. I suppose that you thoroughly comprehend the whole theory of septic influences now, and have made a study, more or less complete, of bacteriology. If you have not, young man, you will soon be left far behind in your professional race. It is impossible for you to comprehend the modern theories of disease and cure without this knowledge. The surgeon, general or special, who has not studied the Listerian theory, and learned something of the proper aseptic treatment of wounds and lesions, should retire to the woods, for he is entirely unfit to practice any branch of the healing art.

If you have this indispensable knowledge, I need spend but a few moments in speaking of the treatment of common alveolar abscess. It is essentially antiseptic. If a fistula shall have been established through the alveolar walls, the whole theory is very simple, and a few plain directions are sufficient. You must first get unobstructed access to the nerve canal or canals, as mentioned in my last letter to you. Then clean the canals and pulp chamber carefully, and see if the foramen at the end of the root is open. All this should be done carefully, and without the thrusting of any of the offensive debris through the root. Now, after adjusting the rubber dam, force into the tooth and through the whole fistulous tract some reliable antiseptic. For a long time my almost exclusive remedy for this was pure carbolic acid. This is not only an antiseptic, but a cauterant as well, and it will burn out the diseased territory and form the initial point for a new growth of tissue. One thorough application is usually sufficient. If you use this remedy, the rubber dam is essential to prevent excoriation of the lips and gums. Wind a few filaments of cotton upon a very delicate broach, and use this as a piston to pump the remedy in until it appears freely at the fistulous opening. This is usually sufficient, and the canal may be at once filled, though I usually postpone the filling for a week, that I may make assurance doubly sure. This means of treatment is very effective, and is usually attended by no pain.

Of late years, when I am sure that I have the fistulous tract open, I more frequently fill a syringe with peroxide of hydrogen, insert the point into the cavity and pack closely around it with cotton dipped in sandarac, and then force a stream quite through. This is continued as long as there is any sign of effervesence, after which the nerve canals are filled.

If there be no fistulous opening at the gums, but the abscess is discharging through the tooth, the treatment is the same in principle, but it will be more tedious. The nerve canals and the pulp-chamber, after being thoroughly cleansed, must be filled with cotton saturated with the antiseptic, which latter must be forced through the foramen if possible. The treatment must be continued and the cotton changed as often as it loses its antiseptic power, and this you may determine by the smell. For this reason an antiseptic having a distinct odor should be used, and before it loses its peculiar fragrance and becomes offensive it must be changed, even though this implies hourly treatment. All depends upon keeping the cotton aseptic. When this condition is permanently secured the root may be filled.

